# Selections and Abstracts

**Published:** 1906-03

**Authors:** 


### SELECTIONS AND ABSTRACTS.

### Lines Read at a Dinner Given in Honor of Dr. Robert Fletcher, January 11, 1906, Upon His Eighty-Third Birthday.

By William S. Thayer.

If age means but the sum of leaves
  Time's calendars unfold,
Our honored guests must recognize
  That he is rather old.

If youth means elasticity,
  A ready wit and tongue,
A mind alert, a spirit gay,
  He's eminently young.

If age means stores of learning ranged
  On ordered shelves along,
Still crescent 'neath the nurture of
  A guardian sage and strong,

All centered in an index which
  Is hidden in the brain,
Our friend has surely reached an age
  We may not see again.

If youth betrays itself by vim,
  And broken bones soon healed,
A constant tendency to pry
  In every secret field ;

By always leading in the van
  Of life's long search for truth—
Why then, despite his years, he's but
  The prototype of youth !

So here's a glass to fourscore years,
  To ripe and wise old age,
To all the gains which gen'rous time
  Scores on his record page ;

And here's a glass to fervid youth,
  To supple limbs and mind
Wherein hope's rainbow arches o'er
  All doubts that low'r behind ;

And here's a health to him in whom
All these conditions meet,
Old in all virtues born of days,
Young where'er youth is sweet.

Long may he live to taste alike
Of age and youth the joys;
Old, yes, in years, but in his heart
A boy among the boys!

—*Johns Hopkins Bulletin*, *Feb.*, *'06.*

---

### RADICAL CURE FOR CORNS AND BUNIONS.

In the *Clinical Journal* E. H. Freeland has an excellent article on this much neglected topic. He says it is difficult to understand why this disease, which is as troublesome as it is common, should be so neglected by medical men. In consequence it has fallen almost exclusively into the hands of irregular practitioners. The word "corn," when used in medical parlance, signifies a small localized epithelial swelling, appearing commonly on the foot or toe, running a chronic course, giving rise to considerable pain. From a surgical standpoint it may be looked upon as an affair of small moment, but from the viewpoint of the sufferer it is a more serious matter. These tumors render locomotion difficult and sometimes impossible, even unfitting the sufferer for performing his ordinary duties. From time immemorial, corns have been divided into two varieties, the "hard" and the "soft." This in reality is a distinction without a difference, for structurally they are identical. All corns in fact are hard, and the difference is one of superficial appearance only due to position. The so-called soft corns present a sodden, macerated appearance on the surface, owing to the pent-up heat and moisture between the toes, where they commonly exist. When one of these growths is completely severed from its surroundings and examined it shows that the major portion of the growth lies beneath the surface. Corns are usually described as being conical in shape, the apex being directed deeply toward the true skin, while the base projects on the surface. This description, like many others which have crystalized through long ages without verification, is only partially true and by no means holds good for all or even the majority of cases. Corns vary much in shape, determined largely by their degree of maturity and situation. In the initial phases of its development a corn assumes the form of a stunted cone, of which the apex projects on the surface, and the base, presenting a slightly convex outline, rests in the surface of the true skin. In corns of the ordinary type this condition of affairs soon becomes altered or entirely reversed. Still preserving the conical shape the base, which becomes more or less concave, presses on the surface, while the apex, considerably blunted, projects deeply toward the surface of the true skin. The corn seems to penetrate and become embedded in the subcutaneous tissue, carrying the dermis before it. Corns when met with in this stage of their development, have been somewhat fantastically compared to nails driven into a board, hence the terms clavus. As development goes on another change in shape takes place. All old corns tend to assume a discoid shape, and when associated with a bursa become compressed vertically and biconcave. These variations in shape are due to external pressure; the growths, while increasing superficially by the addition of new material from beneath, are forced to expand in the direction of least resistence—that is to say, deeply and circumferencially. The so-called soft corn retains its primitive shape to the end, the superficial cells being shed almost as quickly as they are formed.

A section of a corn shows to the naked eye a fibrous structure marked by numerous striations. In the majority of cases three horizontal layers varying in color and degree of opacity can be clearly made out traversing the entire substance of the corn. Microscopic examination of the stained section shows that all the layers of the epidermis share in the hypertrophy to a variable degree, the increase being most marked in the stratum corneum and stratum mucosum—that is to say, in the deepest layers and most superficial. The pale, horizontal line of variable thickness traversing the corn in a sinuous manner longitudinally corresponds to the stratum lucidum, the layer deeper than this representing the stratum mucosum, and the layer superficial to it the stratum corneum, while the vertical striations, represent columns of cells pressing gradually toward the surface. Certain changes, chiefly of an atrophic nature, are observed in the true skin. The papillæ are compressed and elongated when active growth is going on; squat and stunted where full growth has been obtained. The deeper layers of the skin and subcutaneous tissues are compressed and thin, but contain blood-vessels in abundance. A corn grows in the same manner as normal epidermis, but the multiplication of the cells is more rapid. The cells increase at a greater rate than they are shed. They accumulate into a compact mass at the site of the tumor. Sometimes, and always in the case of "soft" corns, a small fluid-containing sac or bursa forms in the subcutaneous tissue beneath the corn. The function of this structure, and the part which it plays in the life history of the corn, are points which are too frequently overlooked. Originally defensive, acting as a buffer between the under surface of the corn and the underlying bones, it is sometimes converted into a source of danger. It is said that a corn inflames or suppurates, this simply means that the bursa has become inflamed. It is this liability to inflammation of the bursa that gives a corn its chief pathologic gravity. An inflammation of the bursa connected with a corn may extend to joint, bone or tendon. Ordinarily pressure of ill-fitting boots is the cause of corns. Remedying these conditions may give relief, but in some cases it does not. Pressure and friction are undoubtedly causes, but there must be some predisposing factor, as some feet never develop corns, while others suffer excruciating pain from them, while the conditions as to footgear and exercise are practically the same. Notwithstanding the removal of the exciting cause many corns tend to increase. Other methods of treatment aim to diminish pressure by reducing the bulk of the growth. These consist in the application of certain chemical substances or instrumental procedures. In this way the pain is relieved, but permanent relief is not obtained. In order to determine how much of the growth could be removed, Freeland soaked several corns in a saturated solution of salicylic acid and found that separation invariably took place at the junction of the stratum corneum and the stratum bicidum—or in other words, the horny layer was exfoliated.

Instrumental procedure as practiced by chiropodists only removes the horny layer. This partial removal is only palliative, and if a radical cure is to be effected the entire growth must be excised. If the bursa is left behind it frequently becomes inflamed. The operation may be done under general or local anesthesia. If the latter, 5 minims more or less of a 4 per cent. solution of eucaine is injected into the subcutaneous tissue beneath the corn. Waiting a few minutes, the superficial parts of the site of the incision are made insensible by ethyl chloride. Two semilunar incisions are made around the circumference of the growth, taking care that they penetrate well into the subcutaneous tissue. One or two sutures are introduced and an antiseptic dressing applied. Corns are frequently associated with some common deformities of the foot, and when present in such cases must be regarded as the effect rather than a cause of trouble. Under these circumstances the treatment of corns must be the treatment of the deformity. Never excise a corn until a careful examination of the foot has been made to determine whether there exists any deformity to reasonably account for its presence. The term bunion is used to denote a painful swelling which occurs on the inner aspect of the ball of the great toe. The first stage of a bunion consists of a partial dislocation outward of the great toe, which renders the head of the metatarsal bone unduly prominent. As the time goes on the exposed head of the metatarsal bone enlarges from periostitis, and the patient begins to experience pain and discomfort. As the case advances further a bursa forms over the inner aspect of the bony boss. The pain then becomes more intense, and there are exacerbations due to acute attacks of inflammation in the bursa and frequently in the joint, with which the bursa often communicates. Matters are often further complicated by a corn which forms on the summit of the bursa. The condition of the patient is then miserable in the extreme, constant pain and permanent lameness being the result. This condition of hallux vulgus usually begins in childhood, but rarely gives rise to serious trouble until adult life. In the majority of cases the cause is purely mechanical and may be traced to wearing of ill-fitting boots, which are too narrow and bunch the toes. The pressure gradually forces the big toe outward, the head of the metatarsal bone being rendered more prominent and more exposed to injury. Friction results in subacute periostitis, and once set going the inflammatory process persists and becomes chronic, more especially in those of a gouty or rheumatic diathesis. There is a progressive enlargement of the head of the bone. In the treatment of the affection, cases that are seen early, where there is only dislocation of the toe, may be remedied by mechanical appliances, but as a rule relief is seldom sought until symptoms in shape of pain and lameness due to the inflammatory changes have developed. Even if the appliances can be tolerated, it is manifest that straightening the great toe will not check the inflammatory changes, which are at the root of the evil. Many cases if seen early may be relieved by wearing boots constructed on proper principles. The soles should be broad and the uppers fit well at the instep and be roomy in front and well blocked out at the toe. This gives room for the toes to spread, and the enlarged joint is relieved from irritation. So far as the relief of symptoms is concerned, the displacement of the toe may be ignored. Where properly fitting boots do not relieve the trouble, then more radical procedures must be employed. Symptoms only arise when the head of the bone enlarges from inflammatory changes. The formation of a bursa and corn is what causes the pain, and these are only gotten rid of by a reduction in the size of the bone, which by pressure gives origin to these troubles. In these cases excision of the head of the metatarsal bone obviates the mischief. The joint cavity which has been subject to chronic inflammation is obliterated, and the metatarsal bone is joined to the base of the first phalanx by a band of fibrous tissue which gives a functionally useful joint. If the bursa is small it can be ignored. If, however, it be large and has a thick wall it should be removed, while the corn, if present, should always be excised.

While Freeland shows the inadequacy of the ordinary palliative measures in dealing with corns, the fact remains that a very large proportion of them may be readily relieved by such means if the treatment is persistent. As he points out, a corn is at first small and conical, then by pressure it gradually broadens and may become a tumor of considerable proportions. It is the pressure which leads to the development of a bursa. This once inflamed, or if it have communication with a sheath of a tendon or the periosteum, is almost incurable without surgical intervention. But even in severe corns, if the cuticle is removed down to the septum lucidum by applications of salicylic acid or other chemical means and the pressure is relieved, and if this is continued for weeks, the corn gradually becomes smaller and may disappear. The possibility of an ultimate success with the so-called palliative treatment is much brighter than would have been supposed from a study of Freeland's article. The usual treatment which a corn receives is to have the cuticle removed either by mechanical or chemical means and then wait until the corn is again painful before anything further is done. Such a method of treatment is rarely successful. The regular removal of the horny layer by chemical means as often as twice a week, if persisted in for a considerable period, frequently results in a cure of aggravated examples of the affection.—*Pacific Medical Journal*, February, 1906.

---

### OPERATION FOR THE RADICAL CURE OF INGUINAL HERNIA UNDER COCAINE ANESTHESIA.*

Statistics of various countries show that from ene-eighth to one-sixteenth of the world's population suffer from hernia, and the vital statistics of the Census show that one out of every six hundred people who die, in the United States, die from hernia. It is, therefore, apparent that hernia is one of the most common and serious disabilities of mankind, and every practitioner should familiarize himself with the recent advances that have been made in its treatment, in order to do justice to his patients.

Hippocrates described the different varieties of hernia, and Celsus wrote with great minuteness in regard to the treatment of the strangulated variety. He advised opening the hernia sac, dividing the constricting ring, and returning the intestines to the abdominal cavity.

---

*Read by Stuart McGuire, M.D., Richmond, Va., President, and Professor of Principles of Surgery and Clinical Surgery, University College of Medicine, Richmond, Va., Surgeon to St. Luke's Hospital, etc., before the thirty-sixth annual session of the Medical Society of Virginia, held at Norfolk, Va., October 24-27, 1905.

In the middle ages many operations were done for reducible hernia, the most popular being castration and the obliteration of the hernial opening. The mortality, as well as the number of failures to effect a cure, brought the procedure into well-merited disfavor, and, in 1804, we find Sir Astley Cooper condemning any operation except in cases where strangulation threatened life.

With the introduction of anesthetics and antiseptics, however, conditions were absolutely changed, operations formerly painful and dangerous were rendered painless and free from risk, and surgeons at once began to operate, especially on inguinal hernia, it being the variety most commonly seen. Much to their surprise, it was found that while the patients did not die from the operation they were not cured by it. In one-half the cases there was shortly a recurrence of the trouble, and the patient's last condition was frequently worse than the first. It was at length appreciated that the fault was in the method of operating; in other words, that it was not sufficient to resect the sac and constrict the external ring, but for success it was also essential to open the inguinal canal and constrict the internal ring. One of the first operations based on this principle was that of Bassini, an Italian surgeon. It soon grew in popularity and was adopted by the profession throughout the world. As the steps are familiar to all surgeons, I will not describe them here. With the modifications suggested by Fergusson, of Chicago, and Mayo, of Rochester, it is now one of the most satisfactory procedures in surgical practice. Its mortality is practically nil, and the results are over 95 per cent. of permanent cures. The surgeon who twenty, or even ten, years ago hesitated to advise a man with reducible inguinal hernia to submit to an operation, on account of the danger to life and the uncertainty of permanent cure, now confidently urges an operation as a safe and certain relief from the irksome necessity of wearing a truss and the actual daily danger of possible strangulation.

Despite this attitude of the profession patients have been found slow and reluctant to profit by it, and an army of truss fitters are still actively at work. In discussing the operation with patients I have invariably found that the one and insuperable objection was the dread of a general anesthetic. I was therefore delighted when, at the last meeting of the Tri-State Medical Association in Greensboro, N. C., to meet Dr. J. A. Bodine, of New York, and see him operate on two cases ot inguinal hernia with local anesthesia. I had, in common with the rest of the profession, read Dr. Bodine's paper on the subject, but until I found for myself the ease and effectiveness of the method, I did not appreciate its great practical application.

In the last few months I have operated with cocaine anesthesia on ten cases of inguinal hernia, one of them double, and, from my personal experience, I fully agree with Dr. Bodine that cocaine should be employed in all cases of inguinal hernia, unless there are special indications for the use of a general anesthetic. In nine of my cases the patients chatted with the nurses and complained practically of no pain; in one case the patient made a great outcry, but afterwards admitted that he was more scared than hurt, and said that if he had to go through with the operation again he would not take chloroform. In all ten cases the wound healed by primary intention, and, as far as I know, the patients are all permanently cured.

In the evolution of the modern operation for hernia there are different epochs, and a different name is associated with each.

1st. The study of the anatomy of the structures involved, by Cooper.

2d. The discovery of general anesthesia, by Long.

3d. The introduction of antiseptics, by Lister.

4th. The education of the profession to the use of absorbable sutures, by Marcy.

5th. The publication of a method of constricting the ring and canal that effected a permanent cure, by Bassini.

6th. The local use of cocaine in a way to render the operation painless.

Before giving credit for the last, but not the least step, to Bodine, I have investigated his claims to the right. To Crile and Corning belong the credit of showing the value of "blocking" a nerve by injecting its trunk. To Cushing belongs the credit of writing up the anatomy of the nerve supply to the parts involved in inguinal hernia and advising local anesthesia in cases where a general anesthetic was contraindicated. But to Bodine, of New York, unquestionably belongs the credit of popularizing the method, improving the technique and making it practicable in all cases where idiosyncrasy and complications do not exist.

I have not the time, nor is this the place, to describe in detail the technique of the operation. The essential features are the employment of a freshly made one-fourth of one per cent. solution cocaine; the intracuticular injection of the skin in the line of incision; the location of the hypogastric branch of the iliohypogastric; the inguinal branch of the ilio-inguinal, and the genital branch of the genito-crural, and the blocking of these nerves by the injection into their trunks of one or two drops of the solution. I do not believe the operation can be satisfactorily done by the novice, but to the surgeon who knows his anatomy and has had experience in the special operation, the work presents no difficulties. In fact, the operation is easier to do under local than under general anesthesia, because the patient is conscious and able to distend the sac by coughing, so as to facilitate its location and separation.

To use the idiom of our Western friends, we now have a very attractive "*proposition*" to present to a patient who consults us for hernia. We can tell him he can be cured—

1st. Without appreciable danger.

2d. Without much question of permanency.

3d. Without more than two or three weeks detention from business.

4th. Without a general anesthetic.

5th. Without pain.

—*Virginia Medical Semi-Monthly, Feb'y 9, 1906.*

---

SYPHILITIC nasal ulcers may occur anywhere in the nares. They are either superficial or deep and serpiginous with an inflammatory zone. Dry, greenish, offensive crusts appear in abundance. Caries may perforate the septum on the floor of the nose. By way of local treatment Levy directs to cleanse with a warm solution of potassium permanganate, and apply tincture of iron with a cotton applicator.—*Denver Med. Times.*

THE JOURNAL OF THE AMERICAN MEDICAL ASSOCIATION

Is the official organ of the American Medical Association. That association is practically composed of members of all the State Societies in the Union. Every member of that association who pays his membership dues ($5) is entitled to annual subscription. The same price is charged to those not members. In round figures, there are about thirty-five thousand members of that association—including a number of homeopaths, eclectics, etc., who are allowed to join by the present "reorganized" rules of the association. This membership of the National Association guarantees an annual income of about $175,000—*in addition* to an unknown income from advertisements in every issue of the weekly *Journal*, which unknown quantity, we presume, may be estimated at not less than $75,000—thus securing a total annual income of $250,000, or more. How much of this is profit we do not know; possibly $100,000. Such an amount of profit results that, not content with its own success, it is now venturing into the field of competition with other heretofore solid enterprises, such as that of making a directory of the profession of the United States, etc. We can not believe that it was ever the purpose of this American Medical Association to assume the role of such competitions.

The *Journal* is the property of the American Medical Association. It should be the exemplar of ethics in the journalistic field, as well as its dealings with the medical profession. It would seem, therefore, that it would place itself above the plane of ordinary competition with other reputable medical weeklies of the country, and avoid invidious distinctions or comparisons. Instead of adopting, however, this higher ethical course, it puts itself in comparison—both as to the total number of subscribers and amount of matter annually in its columns—with some of the most reputable medical weeklies of the country, as if to show their relative weakness in these respects. Such a course is not dignified—is not becoming—for the obvious intentional effect is to exalt the one and pull down the others. We do not believe that the better element of the profession will sustain the management of the *Journal of the American Medical Association* in any such effort.

The present plan of organization of the American Medical Association has sufficiently injured enough formerly good journals by causing them to limit their spheres of subscription patronage to the State Society memberships of which they have become the so-called official organs or bulletins. The *Journal of the American Medical Association* has secured, through the influences of the State Societies, the salaried drummers it can easily afford to send out under the title of "organizers," etc., a patronage sufficient to let these larger medical weeklies alone in quietly fulfilling their mission as regular medical journals of high merit, and as exponents of independent views.

If the American Medical Association is annually adding materially to its surplus fund by virtue of its increasing membership, there are many fields open for investment, profitable to the profession and to humanity, without undertaking to pull down enterprises that were appreciated and fairly profitable. There are many matters for scientific investigation which the individual doctor has not the means to attempt without financial assistance. From time to time, as circumstances may allow, sufficiently salaried commissions might be organized among qualified workers in the profession to ferret out the truth of reasonable suggestions in some department of medicine. Such a course would be more in keeping with higher professional sentiment than undertaking to pull down enterprises of established value.

Many other avenues of profitable expenditure of the profits of the Association than that of entering into competition with useful business enterprises by distinct corporations might be suggested—were it the purpose of this note to do so.

We do not by any means oppose the American Medical Association. On the contrary, properly conducted, it has a legitimate field of usefulness that no other association of the profession can fill. It is only as a caution that it should cease to antagonize the honest convictions and right of a great mass of the loyal members of the profession that we write. Otherwise, the time will come, as with some of the insurance corporations of the day, when searching inquiries will be made which may threaten the downfall of the now mighty organization.—*Editorial Virginia Medical Semi-Monthly, Feb. 9th, 1906.*

---

### EDUCATION IN SEXUAL SUBJECTS.

T. C. Valentine concludes an interesting paper (*New York and Phil. Med. Jour.*, Feb. 10, 1905), on education in sexual subjects, as follows:

Sexual physiology and hygiene need not be formally taught girls, save in the exceptional instances in which the genesic impulse is prematurely developed.

Sexual physiology and hygiene should be taught every boy, when mental and sexual puberty make him capable of beneficially utilizing the knowledge.

The nature and scope of instruction on sexual subjects should be regulated according to each pupil's ability properly to appreciate the warnings inseparable therefrom.

The age at which a person may safely be instructed in sexual subjects is that age at which, in each individual case, such instruction becomes necessary for the purpose of moral and physical prophylaxis.

The individuality of the parent, physician or teacher should be the guide to the choice of one or the other as the exponent of the facts.

Educational institutions may be utilized for instruction in sexual subjects, but such instruction must be given to small groups of pupils selected because of their mental parity as nearly as may be.

Text-books on elementary hygiene should not contain chapters on sexual physiology. Those charged with imparting instruction on sexual subjects should be provided with separately printed chapters on the physiology and hygiene of these matters. These separately printed chapters could then be given with the greatest discretion to such pupils only whose mental development would preclude their misusing the information derived therefrom.

All instruction to the laity on sexual subjects should be directed essentially to serve as a groundwork for the following ideas:

(a) Many learned men hold that ante-nuptial coitus is not necessary for the health of the individual.

(b) Continence reduces the sexual desire.

(c) Gratification of the sexual impulse before marriage degrades the moral tone and exposes to serious infection.

(d) Venereal diseases are not disgraceful infections, but the result of unfortunate lack of self-control.

(e) The greatest danger at the inception of venereal diseases is in their being maltreated by quacks.

(f) If a person is so unfortunate as to contract a venereal disease, self-preservation should cause him to immediately seek the advice of his family physician.

---

### CHEWING GUM IN TREATMENT OF HYPERCHLORHYDRIA.

Mennier (*Presse Med. Paris*) has been traveling in this country and he informs his countrymen that one in every four American women is chewing gum constantly, and one out of every two men is chewing tobacco or gum. This constant chewing, he was told, was to aid digestion, and he has noticed that the persons chewing presented evidences of hyperchlorhydria. This affection is common among Americans, he adds, as they eat quantities of meat, drink a great deal, and are so devoted to business that they have little time for mastication of their food. On his return home Mennier began to study whether this chewing gum custom was a mere fad or whether it was really beneficial. In hyperchlorhydria the digestion of starch in the stomach is checked by the presence of an excess of hydrochloric acid, and, on the other hand, the presence of the undigested starchy matter induces excessive secretion of the hydrochloric acid. In both of these phases the indications are to help digestion of the ingested starch, and this is what chewing gum accomplishes. If a person chews gum for an hour and ejects the accumulated saliva into a graduated recipient, from 100 to 150 c.c. of saliva will be found in the vessel. In chewing 100 gm. of bread only 15 to 20 c.c. of saliva is secreted and swallowed with the bread. Experiments with the Ewald test breakfast confirmed this theoretical reasoning and showed that chewing gum or some similar substance after a meal has a marked influence on the digestion of starch, promoting it by the large supplementary amounts of saliva swallowed, thus eliminating the second phase of hyperchlorhydria. He now orders chewing gum for all his patients of this class.—*Jour. A. M. A., February 3, '06.*

---

### SHOULD THE YOUTH OF THIS COUNTRY BE INSTRUCTED IN A KNOWLEDGE OF SEXUAL PHYSIOLOGY AND HYGIENE?

Prince A. Morrow (*American Medicine, January 13, 1906*) says that the general principle is laid down that the education of the public is the most valuable of all measures for the prevention of communicable diseases. Its importance is emphasized in the case of diseases the communication of which lies entirely within the control of the individual. The object of the proposed education is to give the youth of this country a clear comprehension of certain physiologic truths which have a direct bearing upon the regulation of their sexual lives, and of the serious consequences in the shape of disease and death which follow a breach of hygienic laws. In other words, it is to teach them how to live according to the laws of a healthy nature. This instruction in the physiology and hygiene of the sex function should form an essential integral part of the education of youth.

Dr. Morrow criticizes our present educational system, the policy of which is to launch the young into the world in complete ignorance of everything pertaining to the laws of life reproduction. In seeking this knowledge the youth is but obeying a law of his mental evolution. Since this knowledge can not be had from legitimate sources—from parents and instructors—it is gained surreptitiously, and usually from depraved sources, dissolute companions or erotic or quackish literature. To be salutary as a safeguard, therefore, this hygienic education should be given in youth, for it is during this period that the foundations of what may be termed the "sexual character" are laid, and habits of mind and practices are formed which in a great measure determine the future sexual life of the individual.—*Med. Age, February 10, '06.*

### THE PHYSIOLOGIC AND LEGAL STATUS OF THE FETUS IN UTERO.

Sanders urges that birth should date from the union of the male and female elements and not from the delivery of the fetus. In formulating the advantages to be derived from such nomenclature he says:

It would be essentially and scientifically correct to locate birth where and when it actually occurs, in spite of the fact that several months would usually elapse before it could be known that a birth had certainly taken place.

It would place a pregnancy, from the first day of its probable occurrence to its termination, on the high legal and moral grounds it deserves to occupy. It would dignify the position of the fetus in utero, and would establish beyond all doubt or confusion the right of the fetus to the same protection, moral and legal, as is accorded to human beings who have completed the period of their uterine existence.

It would unify the terms, fluxion, abortion and miscarriage, under the one term, premature delivery; then, two expressions, premature delivery and delivery at full term, would cover the entire subject.

It would enable lawmakers to enact clear and definite laws for the protection of the fetus in utero, which laws, jurists and juries could administer without doubt or confusion.

It would have a strong tendency to promote virtue and to prevent crime, and to build up in every community a positive demand for the protection of human life at its tenderest and most helpless period; it would tend to educate the people on a subject in reference to which they stand in great need of education and would thereby save the lives of many innocent and undelivered babes.

In concluding he submits the following propositions:

1. The conjunction of male and female germs constitutes, from a scientific standpoint, birth.

2. The term conception should be abolished and that of birth substituted therefor.

3. In dealing with all stages of pregnancy, even the earliest, physicians should realize the extreme gravity of the condition, and should never condemn to death a fetus, however young, without the maturest consideration, and without calling to their aid the highest professional authority within reach; in a word, without carrying the case to the nearest and wisest medical supreme court accessible.

4. The principles herein contended for should be impressed on the members of the profession, taught to medical students and promulgated widely among the people.

5. Medical men should interest themselves to see that the statutes of their respective states are ample for the protection of the fetus in utero.—*Jour. A. M. A.*, Feb. 24th.

---

### ASSISTANT WANTED.

Because of the rapid growth of my practice and drug-store, I am compelled to have an assistant. Would prefer to sell one-third or one-half interest to the right man. Address, with full particulars of experience, Box 88, Cave Spring, Ga.

---

### THE TREATMENT OF CRUSHED HANDS.

Fairlie-Clarke (*The Practitioner*, December, 1905) considers injuries to the hands to be amongst the most important with which the surgeon has to deal. The general principles of treatment are: cut little, stitch little, drain freely, wash freely. The knife is rarely needed in the treatment of crushed hands. Set primary amputations are almost always bad surgery. Trimming is best done with scissors. It is best to render the limb bloodless by means of a tourniquet. It is never necessary to ligature digital vessels, as bleeding will be controlled by pressure dressings. Gauze makes a very good dressing, but if much skin has been lost boroglyceride on lint or moist lint covered with jaconet is to be preferred, for it sticks less to the raw surface when the dressing has to be changed. Parts must be brought together without tension and the fewest possible stitches used. At first the limb should always be placed on a splint. It should either lie upon a pillow at the patient's side or across the chest, or be swung by means of a pulley from the ceiling. Free drainage is essential, and can be accomplished with gauze strips lightly tucked in. In some cases rubber tubes will serve a better purpose. Washing the injured part is important. It is well to have the hand and arm bathed for half an hour night and morning for the first week. The dressing should be such as will not stick. Dressings of this character are boroglyceride on lint, red lotion on lint, carbolic oil on lint.

In injuries of the fingers the greatest care should be taken to avoid amputation. A badly deformed finger is often better than none at all. The secret of success in injuries to the palm lies in free drainage. A limb kept long on splints becomes very stiff. To avoid this movements of the fingers and massage must be employed. If a finger is to be permanently stiff it would be better fixed in the semiflexed position. If a cellulitis has set in, two or three incisions three or more inches in length should be made. Free drainage being secured the arm must be perseveringly used, and the patient encouraged to keep the hand in the bath as much of the day as possible.—*Therapeutic Gazette*, Feb. '06.

---

### THE DIETETICS OF DISEASES OF THE STOMACH.

Hutchinson (*The London Practitioner*) mentions the fact that when the dilatation is slight and of the atonic type, without true pyloric obstruction, the meals should be small, dry, and mainly of animal constituents. The atonic stomach deals only with difficulty with a mixture of liquids and solids, consequently the patient must not drink with his meals. The necessary amount of liquid may be made up by taking sips of hot water between times. All saccharine articles should be avoided, and the starchy constituents restricted to rusks, torrefied toast, or pulled bread; thus the production of flatulence is reduced to a minimum.

Should there be actual obstruction at the pylorus, with stagnation of the contents, one must begin by cleansing the stomach by thorough lavage, which may be repeated daily, and the diet should consist largely of milk, peptonized if necessary, and administered frequently and in small amounts. The milk may sometimes be enriched by one of the concentrated proteid foods, and a limited quantity of starchy food in the form of biscuits or rusks may usually be allowed with safety.

Patients with pyloric obstruction and copious vomiting, or in whom lavage is being carried out, are very apt to suffer from a deficient supply of fluid to the tissues, and much of the cachexia and wasting, which they exhibit, is due to this cause. In such cases the supply of fluid should be supplemented by the administration of water by the bowel, a pint of normal saline being injected night and morning. If this be omitted, the excretion of waste products by the kidneys is apt to become interfered with and uremic symptoms may supervene.

---

### OUTDOOR LIFE VS. CONFINEMENT IN TREATMENT OF BONE TUBERCULOSIS.

On concluding his paper on "Outdoor Life vs. Confinement in the Treatment of Bone Tuberculosis," Augustus Wilson says:

The results of outdoor life that have been observed are: Osseous tuberculosis does not demand the varied atmospheric conditions that are necessary in the treatment of phthisis. Sleeping in tents or shacks with the temperature almost at zero F. has not been injurious. Environment, mechanic fixation, appropriate clothing, feeding, occupation, exercise, massage, Bier's congestive method, various operative and corrective measures require consideration in connection with outdoor life.

The changes noted in patients have been increased circulatory activity, promoting absorption and repair. Increased appetite. Shortening of the period of treatment. Early arrest of the progress of the disease. Less destruction and consequent deformity. Happy, buoyant, hopeful expression showing the markedly improved mental attitude with its decided beneficial influence upon the physical condition. Marked improvement in the general health and strength. Increased vital resistance. Activity and consequent healthy fatigue induces prolonged recuperative sleep. No condition of the patient has been found too serious for betterment. The earlier recourse is had to outdoor life, the better the results. Medicines have been found to be of little use and in no way a substitute for open-air treatment. Surgical procedures should be ultra-conservative and afford the least possible interference with outdoor life. A poor substitute for outdoor life can, when necessary, be provided in cities. Patients with bone tuberculosis can not be expected to completely and permanently recover if they are kept in confinement.—*Penn. Med. Jour.*, Jan. '06.

---

### LUPUS OF THE NOSE CURED BY X-RAYS.

Dr. Du Bois, of Geneva, reports (*Revue medicale de la Suisse Romande,* November 20, 1905) a very remarkable case of a girl, eleven years of age, who, following an attack of measles two years previously, had had a slowly developing tubercular, granulating lesion of the nose. This began at the upper border of the right nostril, and at the time of treatment had extended over the lower half of the nose on both sides. Treatment was begun by applying a wet disinfecting compress to clean off the surface, and this was followed by exposure to X-rays. The bulb was placed at a distance of 15 centimeters. The applications were made on the 1st, 4th, 9th and 17th of May, at which time improvement was noticed. The treatment was continued at five days' interval. In another month there had been considerable diminution of the volume of the lesion. The swelling had disappeared, and new epidermis made a covering for the greater portion of the affected surface. During the following month no applications were made, but the process of repair continued. Late in August and early in September, two more exposures were made of a small surface on the inside of the nostrils. At the time of the report, in November, the cure appeared to be complete, both of the skin and mucous surfaces. Two illustrations from photographs showing the face of the child before and after treatment accompany the article. The cure appears perfect. In all fourteen seances were given. Should any disposition on the part of the disease to return be manifested, the reporter is quite confident that it can be fully checked by the same treatment.

---

PUNCTURED wounds about the knee should be treated with the greatest solicitude and attention to asepsis, in order to prevent infection of the joint.

### AMPUTATIONS BELOW THE KNEE.

In concluding his paper Clapp says:

In making amputations of the leg, there are a few important details which we should not lose sight of:

Be certain of sufficient flap to properly cover end of bone, regardless of how close this may come to the knee joint. Flap must be considered first, length of stump second.

The most uniform good results are obtained by making the long anterior with short posterior flap, bringing the scar well away from end of stump.

Redundancy is always undesirable.

When the length of stump is at the discretion of the operator, it should be from six to nine inches below lower border of patella.

Periosteal flap with coaptation of muscles over end of bone is always desirable.

Always cut the fibula one inch shorter than the tibia and when the amputation is near the knee joint, disarticulate and remove the fibula.

In all amputations, nerves should be drawn out and cut as short as possible.

---

### ARMY MEDICAL CORPS EXAMINATIONS.

Preliminary examinations for appointment of Assistant Surgeons in the Army will be held May 1st and July 31st, 1906, at points to be hereafter designated.

Permission to appear for examination can be obtained upon application to the Surgeon-General, U. S. Army, Washington, D. C., from whom full information concerning the examination can be procured. The essential requirements to securing an invitation are that the applicant shall be a citizen of the United States, shall be between twenty-two and thirty years of age, a graduate of a medical school legally authorized to confer the degree of doctor of medicine, shall be of good moral character and habits, and shall have had at least one year's hospital training or its equivalent in practice. The examinations will be held concurrently throughout the country at points where boards can be convened. Due consideration will be given to the localities from which applications are received, in order to lessen the traveling expenses of applicants as much as possible.

In order to perfect all necessary arrangements for the examinations of May first, applications must be complete and in possession of the Surgeon-General on or before April first. Early attention is therefore enjoined upon all intended applicants.

There are at present twenty-five vacancies in the Medical Corps of the Army.

---

### THE METRIC SYSTEM IN MEDICINE.

A study of our domestic medical literature indicates a continued effort to foist upon us a method whose utility is questionable. While fully appreciating its several advantages, we still maintain that it is not adapted to the needs of the profession in this country—that it can never come into general use, and for this reason might lead to confusion, if not disaster, in the prescribing and dispensing of drugs. In support of this assertion, let the physician, after due study of the system, write out, offhand, a recipe for a three and a half ounce mixture containing small fractions of a grain to the dram or for tablets, each representing a 50th or 75th of a grain of the alkaloids, and the result will be found anything but satisfactory either to the druggist or the doctor.

It seems presumptuous to require the busy physician to give up a method which is effective and thoroughly understood for another which requires much time and practice to acquire and which, when finally mastered, can render our medical work little if any more satisfactory.—*N. E. Med. Journal.*

---

THE examination for tubercle bacilli in the urine by the ordinary method of staining is not decisive by any means, even if the bladder has been catheterized and differential stains for smegma bacilli have been employed. Numerous examinations with the aid of these procedures must be made, and even then the diagnosis is only a presumptive one. The only sure test is by injecting a large quantity of the sediment into a guinea-pig.—*American Journal of Surgery.*

### A New Hemoglobinometer.

Absolutely accurate instruments for measuring the amount of coloring matter present in the blood are too complicated and expensive for the practicing physician, and the simpler instruments are usually so inaccurate that no two give the same readings. A very ingenious hemoglobinometer has been devised by P. Gruetzner (*Münich Med. Woch.*). The principle is the same as in the Fleischl, except that the wedge is formed by the diluted blood, which is compared with a standard color. The wedge is moved up or down until the two colors correspond accurately. Two extra slits above and below will greatly facilitate the matching of colors, and artificial light is not required.—*Med. News.*

---

### Fleas a Source of Infection of Epidemic Cerebrospinal Meningitis.

Verrill says that mosquitoes and the common house-fly have been assailed as carriers of disease, and that the place which fleas may hold is worthy of study. He suggests their relation to the spread of infection of the type attributed to *Diplococcus intracellularis* of Weichselbaum. Fleas must be feared by healthy individuals not alone for sentimental reasons, but because they may play a part in the life cycle of the microorganism mentioned.

---

Diabetes in railroad employees has been studied by Navarre (*Semaine Med.*). On the line from Lyons to the Mediterranean 200 cases were reported by the medical officers of the road—an average of three per 1000. The proportion is much higher among engineers and conductors than among other employees. Navarre concludes that vibrations and jars evidently favor the development of diabetes.—*The Med. Times.*

---

It is not sufficiently established that the character of the crystals found in the urine indicates the presence or identity of lithiasis in the urinary tract. When cystin crystals are constantly found in the sediment, however, if symptoms of lithiasis are present, the stone is probably made up of cystin.—*American Journal of Surgery.*

